# 

**Published:** 1886-09

**Authors:** 


### CONTENTS.

### PROCEEDINGS.

American Dental Association.... 425
SALMAGUNDI .... 474
The Outlook for the Congress.... 475

### EDITORIAL.

American Dental Association.... 476
Southern Dental Association.... 477
Virginia State Dental Association.... 477
Central Illinois Dental Society.... 478

